# Crystal structure of {2,2′-[ethyl­enebis(nitrilo­methanylyl­idene)]diphenolato-κ^4^ *O*,*N*,*N*′,*O*′}oxidovanadium(IV) methanol monosolvate

**DOI:** 10.1107/S1600536814023332

**Published:** 2014-10-29

**Authors:** Rachel E. Hsuan, Jemma E. Hughes, Thomas H. Miller, Nabila Shaikh, Phoebe H. M. Cunningham, Alice E. O’Connor, Jeremiah P. Tidey, Alexander J. Blake

**Affiliations:** a School of Chemistry, The University of Nottingham, University Park, Nottingham NG7 2RD, England

**Keywords:** crystal structure, oxidovanadium(IV), 2,2′-[ethyl­enebis(nitrilo­methanylyl­idene)]diphenolate, *N*,*N*′-bis­(salicyl­idene)ethyl­enedi­amine, hydrogen bonding

## Abstract

Two independent mol­ecules of the title solvated complex, [V(C_16_H_14_N_2_O_2_)O]·CH_3_OH, also known as [*N*,*N*′-bis­(salicyl­idene)ethyl­enedi­amine]­oxidovanadium(IV) or vanadyl salen, crystallize in the asymmetric unit. Each disordered methanol solvent mol­ecule [occupancy ratios 0.678 (4):0.322 (4) and 0.750 (5):0.250 (5)] is linked to a [*N*,*N*′-bis­(salicyl­idene)ethyl­enedi­amine]­oxidovanadium(IV) mol­ecule by an O—H⋯O hydrogen bond and to others by C—H⋯O hydrogen bonds. The resulting extended structure consists of a bilayer of mol­ecules parallel to the *ab* plane. Despite the fact that solvates are common in complexes derived from substituted analogues of the *N*,*N*′-bis­(salicyl­idene)ethyl­enedi­amine ligand, the title solvate is the first one of [*N*,*N*′-bis­(salicyl­idene)ethyl­enedi­amine]­oxidovanadium(IV) to be structurally characterized. The two vanadyl species have very similar inter­nal geometries, which are best characterized as distorted square-based pyramidal with the vanadium atom displaced from the N_2_O_2_ basal plane by 0.5966 (9) Å in the direction of the doubly-bonded oxide ligand.

## Related literature   

The literature reports three structure determinations on the unsolvated title complex, also known as [*N*,*N*′-bis­(salicyl­idene)ethyl­enedi­amine]­oxidovanadium(IV). The first was in the monoclinic space group *P*2_1_/*c* by Riley *et al.* (1986 ▶), the second by Li *et al.* (2004 ▶) in the triclinic space group *P* 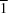 and the third in the monoclinic space group *P*2_1_ (Wang *et al.*, 2008 ▶). All three determinations were carried out at ambient temperature. According to the Cambridge Structural Database (Groom & Allen, 2014 ▶) no solvates of [*N*,*N*′-bis­(salicyl­idene)ethyl­enedi­amine]­oxidovanadium(IV) have been rep­orted previously, although these are common for substituted analogues of the *N*,*N*′-bis­(salicyl­idene)ethyl­enedi­amine ligand.
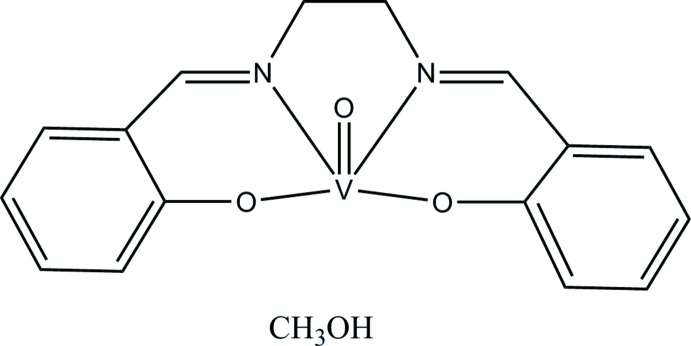


## Experimental   

### Crystal data  

[V(C_16_H_14_N_2_O_2_)O]·CH_4_O
*M* *_r_* = 365.27
Orthorhombic, 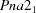

*a* = 12.9597 (4) Å
*b* = 8.8616 (2) Å
*c* = 28.5426 (7) Å
*V* = 3277.92 (15) Å^3^
*Z* = 8
Cu *K*α radiation
μ = 5.27 mm^−1^
*T* = 120 K
0.31 × 0.14 × 0.05 mm

### Data collection  

Agilent GV1000 diffractometer with an Atlas CCD detector
Absorption correction: gaussian (*CrysAlis PRO*; Agilent, 2013 ▶) *T*_min_ = 0.480, *T*_max_ = 0.959
7416 measured reflections
4634 independent reflections
4350 reflections with *I* > 2σ(*I*)
*R*_int_ = 0.025

### Refinement  

*R*[*F* ^2^ > 2σ(*F* ^2^)] = 0.037
*wR*(*F* ^2^) = 0.105
*S* = 1.04
4634 reflections
479 parameters
32 restraints
H-atom parameters constrained
Δρ_max_ = 0.42 e Å^−3^
Δρ_min_ = −0.29 e Å^−3^
Absolute structure: Flack (1983 ▶). 1395 Friedel pairs
Absolute structure parameter: 0.066 (9)

### 

Data collection: *CrysAlis PRO* (Agilent, 2013 ▶); cell refinement: *CrysAlis PRO*; data reduction: *CrysAlis PRO*; program(s) used to solve structure: *SHELXS97* (Sheldrick, 2008 ▶); program(s) used to refine structure: *SHELXL2014* (Sheldrick, 2008 ▶); molecular graphics: *OLEX2* (Dolomanov *et al.*, 2009 ▶); software used to prepare material for publication: *OLEX2*.

## Supplementary Material

Crystal structure: contains datablock(s) I, global. DOI: 10.1107/S1600536814023332/gg2143sup1.cif

Structure factors: contains datablock(s) I. DOI: 10.1107/S1600536814023332/gg2143Isup2.hkl

Supporting information file. DOI: 10.1107/S1600536814023332/gg2143Isup3.cdx

**Fig. 1.**
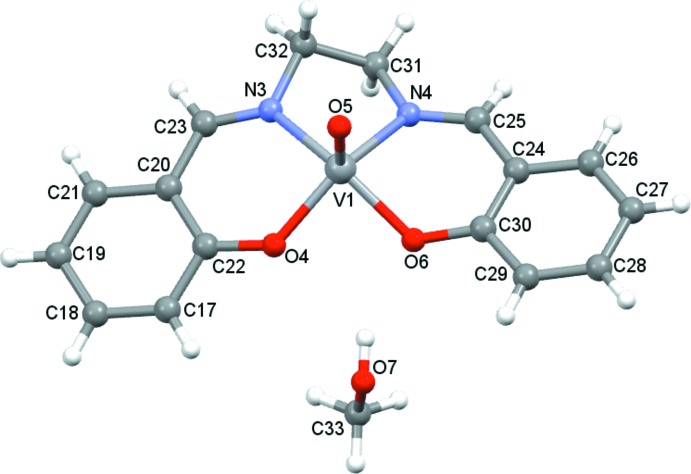
One of the two independent solvated molecules in the asymmetric unit, with atom labels and 50% probability displacement ellipsoids for non-H atoms. Only one component of the disordered MeOH molecule is shown.

. DOI: 10.1107/S1600536814023332/gg2143fig1.tif
One of the two independent solvated mol­ecules in the asymmetric unit, with atom labels and 50% probability displacement ellipsoids for non-H atoms. Only one component of the disordered MeOH mol­ecule is shown.

CCDC reference: 1030592

Additional supporting information:  crystallographic information; 3D view; checkCIF report

## Figures and Tables

**Table:** 

V1O1	1.9155(17)
V1O2	1.9429(16)
V1O3	1.6070(17)
V1N1	2.0597(19)
V1N2	2.051(2)
V2O4	1.9164(16)
V2O5	1.6089(16)
V2O6	1.9314(16)
V2N3	2.062(2)
V2N4	2.060(2)

**Table:** 

O2V1N2	87.30(8)
O2V1N1	150.78(7)
O3V1O2	105.76(8)
O3V1N2	107.35(9)
O3V1O1	113.39(8)
O3V1N1	102.90(8)
N2V1N1	78.80(8)
O1V1O2	86.60(7)
O1V1N2	138.94(8)
O1V1N1	87.26(8)
O6V2N4	87.20(8)
O6V2N3	150.79(7)
O5V2O6	105.81(8)
O5V2N4	107.66(8)
O5V2O4	113.60(8)
O5V2N3	102.81(8)
N4V2N3	78.63(8)
O4V2O6	86.66(7)
O4V2N4	138.40(8)
O4V2N3	87.25(7)

**Table 2:** Hydrogen-bond geometry (, )

*D*H*A*	*D*H	H*A*	*D* *A*	*D*H*A*
C14H14O8^i^	0.93	2.48	3.368(4)	159
C14H14O8*A* ^i^	0.93	2.48	3.299(7)	147
C25H25O7^ii^	0.93	2.51	3.391(4)	158
C25H25O7*A* ^ii^	0.93	2.66	3.457(9)	144
C13H13O3^iii^	0.93	2.60	3.372(3)	141
C23H23O5^iv^	0.93	2.59	3.364(3)	141
O8H8*A*O2	0.82	2.12	2.926(4)	167
O7H7*A*O6	0.82	2.18	2.940(3)	153
O7H7*A*O4	0.82	2.64	3.246(4)	132
O8*A*H8*AA*O1	0.82	2.18	2.984(6)	166

Symmetry codes: (i) 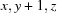; (ii) 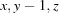; (iii) 
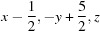
; (iv) 
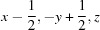
.

## supplementary crystallographic information

### S1. Experimental

Crystals of the title complex were obtained by slow cooling of a methanolic solution of {2,2'-[ethylenebis(nitrilomethylidyne)]diphenolato-κ^4^*O*,*N*,*N*',*O*'}oxidovanadium(IV).

### S2. Refinement

With the exceptions of methyl groups (which were refined as rigid rotating groups) and OH groups (for which were idealized tetrahedral OH were refined as rigid rotating groups), H atoms were allowed to ride on their parent atoms with *U*_iso_(H) = 1.5 × *U*_eq_(C,O) for methyl and OH groups and 1.2 × *U*_eq_(C) for others.

The O8–C34, O8A–C34A, O7–C33 and O7A–C33A distances were restrained to a target value of 1.43 (1) Å. SIMU and RIGU restraints (Sheldrick, 2008) were applied to the solvent molecules, and linked occupancies were used to stabilize the refinement of the MeOH solvent molecules.

For the absolute structure determination, the classical Flack method was preferred over Parsons because the s.u. was lower.

### Crystal data

**Table:**

[V(C_16_H_14_N_2_O_2_)O]·CH_4_O	*D*_x_ = 1.480 Mg m^−^^3^
*M**_r_* = 365.27	Cu *K*α radiation, λ = 1.54184 Å
Orthorhombic, *P**n**a*2_1_	Cell parameters from 3840 reflections
*a* = 12.9597 (4) Å	θ = 5.2–72.3°
*b* = 8.8616 (2) Å	µ = 5.27 mm^−^^1^
*c* = 28.5426 (7) Å	*T* = 120 K
*V* = 3277.92 (15) Å^3^	Block, green
*Z* = 8	0.31 × 0.14 × 0.05 mm
*F*(000) = 1512	

### Data collection

**Table:**

Agilent GV1000 diffractometer with an Atlas CCD detector	4634 independent reflections
Radiation source: Agilent GV1000 (Cu) X-ray Source	4350 reflections with *I* > 2σ(*I*)
Mirror monochromator	*R*_int_ = 0.025
Detector resolution: 10.3271 pixels mm^-1^	θ_max_ = 74.4°, θ_min_ = 5.2°
ω scans	*h* = −15→15
Absorption correction: gaussian (*CrysAlis PRO*; Agilent, 2013)	*k* = −10→6
*T*_min_ = 0.480, *T*_max_ = 0.959	*l* = −33→34
7416 measured reflections	

### Refinement

**Table:**

Refinement on *F*^2^	Hydrogen site location: inferred from neighbouring sites
Least-squares matrix: full	H-atom parameters constrained
*R*[*F*^2^ > 2σ(*F*^2^)] = 0.037	*w* = 1/[σ^2^(*F*_o_^2^) + (0.065*P*)^2^ + 1.252*P*] where *P* = (*F*_o_^2^ + 2*F*_c_^2^)/3
*wR*(*F*^2^) = 0.105	(Δ/σ)_max_ = 0.02
*S* = 1.04	Δρ_max_ = 0.42 e Å^−^^3^
4634 reflections	Δρ_min_ = −0.29 e Å^−^^3^
479 parameters	Absolute structure: Flack (1983), 1395 Friedel pairs
32 restraints	Absolute structure parameter: 0.066 (9)
Primary atom site location: structure-invariant direct methods	

### Special details

**Table:**

Experimental. Absorption correction: CrysAlisPro version 1.171.36.32 (Agilent, 2013). Numerical absorption correction based on Gaussian integration over a multifaceted crystal model.
Geometry. All e.s.d.'s (except the e.s.d. in the dihedral angle between two l.s. planes) are estimated using the full covariance matrix. The cell e.s.d.'s are taken into account individually in the estimation of e.s.d.'s in distances, angles and torsion angles; correlations between e.s.d.'s in cell parameters are only used when they are defined by crystal symmetry. An approximate (isotropic) treatment of cell e.s.d.'s is used for estimating e.s.d.'s involving l.s. planes.

### Fractional atomic coordinates and isotropic or equivalent isotropic displacement parameters (Å^2^)

**Table:**

	*x*	*y*	*z*	*U*_iso_*/*U*_eq_	Occ. (<1)
V1	0.33525 (3)	1.13029 (4)	0.50170 (2)	0.02409 (8)	
V2	0.57988 (3)	0.36777 (4)	0.18892 (2)	0.02364 (8)	
O1	0.24854 (12)	0.95916 (19)	0.49027 (6)	0.0305 (4)	
O2	0.41375 (12)	1.06657 (19)	0.44719 (6)	0.0277 (4)	
O3	0.41104 (12)	1.1086 (2)	0.54575 (6)	0.0298 (4)	
O4	0.49307 (12)	0.53856 (17)	0.20089 (6)	0.0292 (4)	
O5	0.65555 (12)	0.39010 (19)	0.14477 (6)	0.0292 (4)	
O6	0.65825 (12)	0.4296 (2)	0.24318 (6)	0.0294 (4)	
N2	0.35312 (15)	1.3476 (2)	0.47830 (7)	0.0275 (4)	
N4	0.59693 (16)	0.1492 (2)	0.21225 (7)	0.0289 (5)	
C12	0.49325 (19)	1.1394 (3)	0.42862 (7)	0.0283 (6)	
C14	0.42784 (18)	1.3949 (3)	0.45247 (8)	0.0295 (5)	
H14	0.4327	1.4981	0.4471	0.035*	
C30	0.73712 (18)	0.3563 (3)	0.26195 (8)	0.0306 (6)	
C3	0.15814 (17)	0.9193 (3)	0.50810 (8)	0.0280 (5)	
C25	0.6714 (2)	0.1024 (3)	0.23826 (8)	0.0322 (6)	
H25	0.6761	−0.0010	0.2435	0.039*	
N1	0.20834 (14)	1.2335 (2)	0.53104 (7)	0.0269 (4)	
C26	0.8334 (2)	0.1282 (3)	0.28130 (9)	0.0409 (7)	
H26	0.8397	0.0237	0.2803	0.049*	
N3	0.45275 (15)	0.2651 (2)	0.15943 (7)	0.0278 (4)	
C4	0.09719 (17)	1.0158 (3)	0.53631 (7)	0.0273 (5)	
C10	0.6518 (2)	1.1282 (4)	0.38350 (9)	0.0423 (8)	
H10	0.7005	1.0710	0.3674	0.051*	
C8	0.5908 (2)	1.3668 (3)	0.40987 (8)	0.0396 (7)	
H8	0.5986	1.4710	0.4113	0.048*	
C15	0.2266 (2)	1.3957 (3)	0.53910 (9)	0.0326 (6)	
H15A	0.2729	1.4102	0.5654	0.039*	
H15B	0.1621	1.4470	0.5457	0.039*	
C22	0.40338 (16)	0.5789 (3)	0.18283 (8)	0.0273 (5)	
C17	0.36417 (19)	0.7228 (3)	0.19338 (10)	0.0352 (6)	
H17	0.4014	0.7860	0.2132	0.042*	
C24	0.74815 (18)	0.1970 (3)	0.26002 (8)	0.0316 (6)	
C13	0.12347 (18)	1.1718 (3)	0.54445 (8)	0.0295 (5)	
H13	0.0761	1.2316	0.5604	0.035*	
C18	0.2719 (2)	0.7725 (3)	0.17509 (9)	0.0390 (6)	
H18	0.2478	0.8680	0.1830	0.047*	
C1	0.0269 (2)	0.7261 (3)	0.51707 (9)	0.0375 (6)	
H1	0.0032	0.6295	0.5102	0.045*	
C9	0.50432 (18)	1.2990 (3)	0.43126 (7)	0.0301 (6)	
C7	0.6641 (2)	1.2817 (4)	0.38686 (9)	0.0451 (8)	
H7	0.7215	1.3281	0.3737	0.054*	
C20	0.34189 (17)	0.4834 (3)	0.15433 (7)	0.0278 (5)	
C28	0.8976 (2)	0.3678 (4)	0.30590 (9)	0.0440 (8)	
H28	0.9476	0.4251	0.3211	0.053*	
C2	0.11882 (19)	0.7752 (3)	0.49797 (9)	0.0331 (6)	
H2	0.1554	0.7117	0.4780	0.040*	
C23	0.36811 (17)	0.3277 (3)	0.14619 (8)	0.0283 (5)	
H23	0.3205	0.2683	0.1302	0.034*	
C16	0.27463 (19)	1.4566 (3)	0.49437 (9)	0.0333 (6)	
H16A	0.2220	1.4695	0.4705	0.040*	
H16B	0.3064	1.5539	0.5003	0.040*	
C5	0.00379 (18)	0.9625 (3)	0.55506 (8)	0.0323 (6)	
H5	−0.0355	1.0263	0.5738	0.039*	
C21	0.24831 (18)	0.5379 (3)	0.13563 (8)	0.0334 (6)	
H21	0.2088	0.4753	0.1166	0.040*	
C11	0.56715 (19)	1.0573 (3)	0.40398 (8)	0.0344 (6)	
H11	0.5598	0.9533	0.4011	0.041*	
C19	0.21417 (19)	0.6823 (3)	0.14501 (9)	0.0377 (6)	
H19	0.1538	0.7186	0.1315	0.045*	
C27	0.9082 (2)	0.2117 (4)	0.30372 (9)	0.0508 (8)	
H27	0.9650	0.1644	0.3172	0.061*	
C6	−0.03109 (19)	0.8186 (3)	0.54647 (9)	0.0362 (6)	
H6	−0.0919	0.7839	0.5600	0.043*	
C29	0.81364 (18)	0.4385 (4)	0.28575 (8)	0.0360 (6)	
H29	0.8076	0.5429	0.2880	0.043*	
C31	0.51874 (19)	0.0415 (3)	0.19582 (9)	0.0328 (6)	
H31A	0.4662	0.0279	0.2196	0.039*	
H31B	0.5505	−0.0555	0.1896	0.039*	
C32	0.4706 (2)	0.1028 (3)	0.15151 (9)	0.0332 (6)	
H32A	0.5165	0.0879	0.1251	0.040*	
H32B	0.4059	0.0518	0.1451	0.040*	
O8	0.3881 (3)	0.7530 (4)	0.41726 (11)	0.0533 (9)	0.678 (4)
H8A	0.3953	0.8354	0.4299	0.080*	0.678 (4)
O7	0.6257 (3)	0.7431 (3)	0.27352 (11)	0.0573 (9)	0.750 (5)
H7A	0.6295	0.6685	0.2566	0.086*	0.750 (5)
C33	0.5510 (3)	0.7185 (6)	0.30846 (13)	0.0506 (11)	0.750 (5)
H33A	0.4851	0.7011	0.2940	0.076*	0.750 (5)
H33B	0.5469	0.8056	0.3284	0.076*	0.750 (5)
H33C	0.5700	0.6320	0.3268	0.076*	0.750 (5)
C34	0.3009 (4)	0.7570 (6)	0.38743 (15)	0.0614 (14)	0.678 (4)
H34A	0.3138	0.6966	0.3602	0.092*	0.678 (4)
H34B	0.2876	0.8593	0.3781	0.092*	0.678 (4)
H34C	0.2419	0.7180	0.4038	0.092*	0.678 (4)
O7A	0.5715 (10)	0.7560 (9)	0.2706 (2)	0.068 (3)	0.250 (5)
H7AA	0.5107	0.7627	0.2629	0.103*	0.250 (5)
C33A	0.5784 (9)	0.6910 (16)	0.3163 (3)	0.073 (4)	0.250 (5)
H33D	0.6391	0.6293	0.3182	0.110*	0.250 (5)
H33E	0.5185	0.6302	0.3221	0.110*	0.250 (5)
H33F	0.5823	0.7700	0.3393	0.110*	0.250 (5)
C34A	0.3143 (7)	0.7847 (9)	0.37978 (19)	0.041 (2)	0.322 (4)
H34D	0.3315	0.7086	0.3572	0.061*	0.322 (4)
H34E	0.3511	0.8759	0.3726	0.061*	0.322 (4)
H34F	0.2414	0.8038	0.3788	0.061*	0.322 (4)
O8A	0.3422 (6)	0.7342 (7)	0.42510 (16)	0.0481 (19)	0.322 (4)
H8AA	0.3271	0.7990	0.4444	0.072*	0.322 (4)

### Atomic displacement parameters (Å^2^)

**Table:**

	*U*^11^	*U*^22^	*U*^33^	*U*^12^	*U*^13^	*U*^23^
V1	0.02276 (15)	0.02190 (17)	0.02763 (14)	0.00204 (14)	−0.00088 (14)	−0.00101 (14)
V2	0.02314 (15)	0.02248 (17)	0.02531 (14)	−0.00205 (14)	0.00061 (14)	−0.00063 (14)
O1	0.0274 (7)	0.0247 (8)	0.0394 (8)	0.0009 (6)	0.0042 (7)	−0.0034 (7)
O2	0.0263 (7)	0.0274 (8)	0.0295 (7)	−0.0023 (7)	0.0018 (6)	−0.0025 (6)
O3	0.0232 (7)	0.0338 (9)	0.0325 (7)	0.0038 (7)	−0.0038 (6)	0.0015 (7)
O4	0.0276 (7)	0.0246 (8)	0.0355 (7)	−0.0019 (6)	−0.0030 (7)	−0.0032 (7)
O5	0.0249 (7)	0.0318 (8)	0.0310 (7)	−0.0031 (7)	0.0021 (6)	0.0000 (7)
O6	0.0282 (7)	0.0312 (8)	0.0289 (6)	−0.0007 (7)	−0.0021 (6)	−0.0023 (7)
N2	0.0288 (8)	0.0239 (9)	0.0297 (8)	0.0006 (8)	−0.0091 (8)	−0.0023 (8)
N4	0.0326 (9)	0.0250 (9)	0.0290 (8)	0.0013 (8)	0.0067 (8)	0.0004 (8)
C12	0.0333 (11)	0.0334 (12)	0.0183 (8)	−0.0018 (10)	−0.0057 (9)	0.0025 (8)
C14	0.0329 (11)	0.0300 (11)	0.0255 (9)	−0.0055 (10)	−0.0071 (9)	0.0028 (9)
C30	0.0270 (10)	0.0426 (13)	0.0222 (8)	0.0018 (10)	0.0061 (9)	−0.0022 (9)
C3	0.0259 (9)	0.0266 (10)	0.0316 (9)	0.0010 (9)	−0.0035 (9)	0.0032 (9)
C25	0.0432 (12)	0.0287 (11)	0.0248 (9)	0.0079 (10)	0.0086 (10)	0.0029 (9)
N1	0.0230 (8)	0.0278 (9)	0.0301 (8)	0.0034 (7)	−0.0022 (7)	−0.0059 (8)
C26	0.0420 (13)	0.0542 (16)	0.0266 (10)	0.0179 (12)	0.0028 (11)	0.0024 (11)
N3	0.0285 (9)	0.0256 (9)	0.0294 (8)	−0.0043 (8)	0.0036 (8)	−0.0056 (8)
C4	0.0263 (10)	0.0307 (11)	0.0248 (8)	0.0021 (9)	−0.0051 (8)	0.0022 (9)
C10	0.0376 (13)	0.0658 (19)	0.0234 (9)	0.0038 (13)	0.0046 (10)	0.0017 (11)
C8	0.0449 (14)	0.0480 (15)	0.0260 (10)	−0.0143 (12)	−0.0023 (10)	0.0045 (10)
C15	0.0321 (11)	0.0289 (11)	0.0368 (11)	0.0081 (10)	−0.0023 (10)	−0.0079 (9)
C22	0.0228 (9)	0.0292 (11)	0.0300 (9)	0.0006 (9)	0.0034 (9)	0.0061 (9)
C17	0.0319 (10)	0.0290 (11)	0.0447 (12)	−0.0012 (10)	0.0053 (11)	0.0022 (11)
C24	0.0346 (11)	0.0369 (12)	0.0233 (8)	0.0073 (11)	0.0044 (9)	0.0012 (10)
C13	0.0254 (10)	0.0372 (12)	0.0257 (9)	0.0077 (10)	−0.0029 (9)	−0.0041 (9)
C18	0.0391 (12)	0.0319 (12)	0.0458 (12)	0.0081 (11)	0.0133 (11)	0.0098 (11)
C1	0.0357 (12)	0.0302 (11)	0.0468 (12)	−0.0025 (11)	−0.0094 (11)	0.0112 (11)
C9	0.0310 (10)	0.0420 (13)	0.0172 (8)	−0.0051 (11)	−0.0043 (9)	0.0023 (9)
C7	0.0394 (13)	0.0687 (19)	0.0272 (10)	−0.0097 (13)	0.0092 (10)	0.0072 (12)
C20	0.0225 (9)	0.0351 (12)	0.0258 (9)	−0.0038 (9)	0.0030 (8)	−0.0002 (9)
C28	0.0322 (12)	0.075 (2)	0.0250 (10)	−0.0029 (13)	−0.0005 (10)	−0.0022 (11)
C2	0.0306 (11)	0.0276 (11)	0.0411 (11)	−0.0002 (10)	−0.0026 (10)	0.0023 (10)
C23	0.0230 (9)	0.0349 (12)	0.0269 (9)	−0.0054 (10)	0.0001 (8)	−0.0029 (9)
C16	0.0349 (11)	0.0237 (11)	0.0412 (11)	0.0045 (10)	−0.0115 (10)	−0.0012 (9)
C5	0.0268 (10)	0.0438 (14)	0.0262 (9)	0.0024 (10)	−0.0019 (9)	0.0028 (10)
C21	0.0265 (10)	0.0468 (14)	0.0269 (9)	−0.0018 (11)	0.0017 (9)	0.0081 (10)
C11	0.0348 (11)	0.0461 (14)	0.0223 (8)	0.0024 (11)	−0.0023 (9)	−0.0005 (10)
C19	0.0292 (11)	0.0481 (14)	0.0358 (10)	0.0045 (11)	0.0040 (10)	0.0134 (11)
C27	0.0431 (13)	0.079 (2)	0.0301 (11)	0.0202 (15)	−0.0037 (11)	0.0057 (13)
C6	0.0263 (10)	0.0466 (14)	0.0356 (10)	−0.0044 (11)	−0.0024 (10)	0.0126 (11)
C29	0.0291 (10)	0.0553 (15)	0.0238 (9)	−0.0055 (11)	0.0001 (9)	−0.0019 (10)
C31	0.0377 (11)	0.0225 (11)	0.0383 (11)	−0.0061 (9)	0.0077 (10)	−0.0031 (10)
C32	0.0340 (11)	0.0263 (11)	0.0394 (11)	−0.0039 (10)	0.0084 (10)	−0.0086 (10)
O8	0.0693 (19)	0.0407 (16)	0.0501 (15)	0.0048 (15)	−0.0044 (16)	−0.0062 (14)
O7	0.0792 (19)	0.0308 (13)	0.0618 (16)	−0.0094 (14)	0.0178 (16)	−0.0105 (13)
C33	0.055 (2)	0.058 (2)	0.0383 (16)	0.006 (2)	−0.0047 (17)	−0.0122 (16)
C34	0.086 (3)	0.057 (3)	0.0412 (19)	−0.025 (2)	−0.008 (2)	0.0187 (19)
O7A	0.122 (8)	0.028 (4)	0.055 (4)	0.017 (5)	−0.020 (4)	−0.016 (3)
C33A	0.043 (5)	0.105 (9)	0.071 (5)	0.049 (5)	−0.002 (4)	0.009 (5)
C34A	0.076 (5)	0.028 (4)	0.019 (3)	−0.025 (4)	0.012 (3)	0.000 (3)
O8A	0.090 (5)	0.029 (3)	0.025 (2)	0.008 (3)	−0.002 (3)	−0.003 (2)

### Geometric parameters (Å, º)

**Table:**

V1—O1	1.9155 (17)	C13—H13	0.9300
V1—O2	1.9429 (16)	C18—H18	0.9300
V1—O3	1.6070 (17)	C18—C19	1.392 (4)
V1—N1	2.0597 (19)	C1—H1	0.9300
V1—N2	2.051 (2)	C1—C2	1.381 (4)
V2—O4	1.9164 (16)	C1—C6	1.393 (4)
V2—O5	1.6089 (16)	C7—H7	0.9300
V2—O6	1.9314 (16)	C20—C23	1.440 (4)
V2—N3	2.062 (2)	C20—C21	1.410 (3)
V2—N4	2.060 (2)	C28—H28	0.9300
O6—C30	1.324 (3)	C28—C27	1.392 (5)
O2—C12	1.326 (3)	C28—C29	1.381 (4)
N4—C25	1.286 (3)	C2—H2	0.9300
N4—C31	1.469 (3)	C23—H23	0.9300
N2—C14	1.287 (3)	C16—H16A	0.9700
N2—C16	1.476 (3)	C16—H16B	0.9700
O1—C3	1.325 (3)	C5—H5	0.9300
C12—C9	1.424 (4)	C5—C6	1.375 (4)
C12—C11	1.393 (4)	C21—H21	0.9300
O4—C22	1.321 (3)	C21—C19	1.380 (4)
C14—H14	0.9300	C11—H11	0.9300
C14—C9	1.439 (3)	C19—H19	0.9300
C30—C24	1.420 (4)	C27—H27	0.9300
C30—C29	1.406 (4)	C6—H6	0.9300
C3—C4	1.415 (3)	C29—H29	0.9300
C3—C2	1.405 (4)	C31—H31A	0.9700
C25—H25	0.9300	C31—H31B	0.9700
C25—C24	1.442 (4)	C31—C32	1.511 (4)
N1—C15	1.474 (3)	C32—H32A	0.9700
N1—C13	1.286 (3)	C32—H32B	0.9700
C26—H26	0.9300	O8—H8A	0.8200
C26—C24	1.401 (4)	O8—C34	1.416 (6)
C26—C27	1.377 (4)	O7—H7A	0.8200
N3—C23	1.286 (3)	O7—C33	1.407 (5)
N3—C32	1.474 (3)	C33—H33A	0.9600
C4—C13	1.443 (4)	C33—H33B	0.9600
C4—C5	1.405 (3)	C33—H33C	0.9600
C10—H10	0.9300	C34—H34A	0.9600
C10—C7	1.373 (5)	C34—H34B	0.9600
C10—C11	1.393 (4)	C34—H34C	0.9600
C8—H8	0.9300	O7A—H7AA	0.8200
C8—C9	1.411 (4)	O7A—C33A	1.428 (9)
C8—C7	1.379 (4)	C33A—H33D	0.9600
C15—H15A	0.9700	C33A—H33E	0.9600
C15—H15B	0.9700	C33A—H33F	0.9600
C15—C16	1.520 (4)	C34A—H34D	0.9600
C22—C17	1.405 (4)	C34A—H34E	0.9600
C22—C20	1.419 (3)	C34A—H34F	0.9600
C17—H17	0.9300	C34A—O8A	1.416 (7)
C17—C18	1.376 (4)	O8A—H8AA	0.8200
			
O2—V1—N2	87.30 (8)	C6—C1—H1	119.4
O2—V1—N1	150.78 (7)	C12—C9—C14	122.6 (2)
O3—V1—O2	105.76 (8)	C8—C9—C12	118.7 (2)
O3—V1—N2	107.35 (9)	C8—C9—C14	118.6 (2)
O3—V1—O1	113.39 (8)	C10—C7—C8	119.7 (3)
O3—V1—N1	102.90 (8)	C10—C7—H7	120.2
N2—V1—N1	78.80 (8)	C8—C7—H7	120.2
O1—V1—O2	86.60 (7)	C22—C20—C23	122.1 (2)
O1—V1—N2	138.94 (8)	C21—C20—C22	119.7 (2)
O1—V1—N1	87.26 (8)	C21—C20—C23	118.1 (2)
O6—V2—N4	87.20 (8)	C27—C28—H28	119.6
O6—V2—N3	150.79 (7)	C29—C28—H28	119.6
O5—V2—O6	105.81 (8)	C29—C28—C27	120.7 (3)
O5—V2—N4	107.66 (8)	C3—C2—H2	119.4
O5—V2—O4	113.60 (8)	C1—C2—C3	121.2 (2)
O5—V2—N3	102.81 (8)	C1—C2—H2	119.4
N4—V2—N3	78.63 (8)	N3—C23—C20	124.6 (2)
O4—V2—O6	86.66 (7)	N3—C23—H23	117.7
O4—V2—N4	138.40 (8)	C20—C23—H23	117.7
O4—V2—N3	87.25 (7)	N2—C16—C15	108.1 (2)
C30—O6—V2	126.23 (15)	N2—C16—H16A	110.1
C12—O2—V1	125.84 (15)	N2—C16—H16B	110.1
C25—N4—V2	124.78 (18)	C15—C16—H16A	110.1
C25—N4—C31	119.5 (2)	C15—C16—H16B	110.1
C31—N4—V2	115.70 (15)	H16A—C16—H16B	108.4
C14—N2—V1	125.24 (17)	C4—C5—H5	119.1
C14—N2—C16	118.9 (2)	C6—C5—C4	121.8 (2)
C16—N2—V1	115.82 (15)	C6—C5—H5	119.1
C3—O1—V1	131.62 (15)	C20—C21—H21	119.3
O2—C12—C9	122.7 (2)	C19—C21—C20	121.3 (2)
O2—C12—C11	118.8 (2)	C19—C21—H21	119.3
C11—C12—C9	118.4 (2)	C12—C11—C10	121.2 (3)
C22—O4—V2	131.37 (15)	C12—C11—H11	119.4
N2—C14—H14	117.7	C10—C11—H11	119.4
N2—C14—C9	124.5 (2)	C18—C19—H19	120.7
C9—C14—H14	117.7	C21—C19—C18	118.7 (2)
O6—C30—C24	123.4 (2)	C21—C19—H19	120.7
O6—C30—C29	119.0 (2)	C26—C27—C28	119.0 (3)
C29—C30—C24	117.6 (2)	C26—C27—H27	120.5
O1—C3—C4	123.5 (2)	C28—C27—H27	120.5
O1—C3—C2	118.9 (2)	C1—C6—H6	120.8
C2—C3—C4	117.6 (2)	C5—C6—C1	118.4 (2)
N4—C25—H25	117.3	C5—C6—H6	120.8
N4—C25—C24	125.4 (2)	C30—C29—H29	119.3
C24—C25—H25	117.3	C28—C29—C30	121.4 (3)
C15—N1—V1	111.60 (15)	C28—C29—H29	119.3
C13—N1—V1	127.96 (17)	N4—C31—H31A	110.0
C13—N1—C15	120.3 (2)	N4—C31—H31B	110.0
C24—C26—H26	119.2	N4—C31—C32	108.60 (19)
C27—C26—H26	119.2	H31A—C31—H31B	108.4
C27—C26—C24	121.5 (3)	C32—C31—H31A	110.0
C23—N3—V2	127.69 (17)	C32—C31—H31B	110.0
C23—N3—C32	120.6 (2)	N3—C32—C31	106.66 (19)
C32—N3—V2	111.60 (15)	N3—C32—H32A	110.4
C3—C4—C13	122.6 (2)	N3—C32—H32B	110.4
C5—C4—C3	119.7 (2)	C31—C32—H32A	110.4
C5—C4—C13	117.6 (2)	C31—C32—H32B	110.4
C7—C10—H10	119.7	H32A—C32—H32B	108.6
C7—C10—C11	120.5 (3)	C34—O8—H8A	109.5
C11—C10—H10	119.7	C33—O7—H7A	109.5
C9—C8—H8	119.3	O7—C33—H33A	109.5
C7—C8—H8	119.3	O7—C33—H33B	109.5
C7—C8—C9	121.4 (3)	O7—C33—H33C	109.5
N1—C15—H15A	110.5	H33A—C33—H33B	109.5
N1—C15—H15B	110.5	H33A—C33—H33C	109.5
N1—C15—C16	106.33 (19)	H33B—C33—H33C	109.5
H15A—C15—H15B	108.7	O8—C34—H34A	109.5
C16—C15—H15A	110.5	O8—C34—H34B	109.5
C16—C15—H15B	110.5	O8—C34—H34C	109.5
O4—C22—C17	118.7 (2)	H34A—C34—H34B	109.5
O4—C22—C20	123.8 (2)	H34A—C34—H34C	109.5
C17—C22—C20	117.5 (2)	H34B—C34—H34C	109.5
C22—C17—H17	119.2	C33A—O7A—H7AA	109.5
C18—C17—C22	121.5 (2)	O7A—C33A—H33D	109.5
C18—C17—H17	119.2	O7A—C33A—H33E	109.5
C30—C24—C25	121.7 (2)	O7A—C33A—H33F	109.5
C26—C24—C30	119.7 (2)	H33D—C33A—H33E	109.5
C26—C24—C25	118.6 (2)	H33D—C33A—H33F	109.5
N1—C13—C4	124.1 (2)	H33E—C33A—H33F	109.5
N1—C13—H13	117.9	H34D—C34A—H34E	109.5
C4—C13—H13	117.9	H34D—C34A—H34F	109.5
C17—C18—H18	119.4	H34E—C34A—H34F	109.5
C17—C18—C19	121.2 (3)	O8A—C34A—H34D	109.5
C19—C18—H18	119.4	O8A—C34A—H34E	109.5
C2—C1—H1	119.4	O8A—C34A—H34F	109.5
C2—C1—C6	121.2 (3)	C34A—O8A—H8AA	109.5
			
V2—O6—C30—C24	−29.7 (3)	C4—C5—C6—C1	−2.1 (4)
V2—O6—C30—C29	151.31 (17)	C15—N1—C13—C4	177.3 (2)
V2—N4—C25—C24	7.9 (3)	C22—C17—C18—C19	−0.6 (4)
V2—N4—C31—C32	22.2 (2)	C22—C20—C23—N3	−7.4 (4)
V2—O4—C22—C17	−170.69 (17)	C22—C20—C21—C19	−1.0 (3)
V2—O4—C22—C20	11.4 (3)	C17—C22—C20—C23	−172.0 (2)
V2—N3—C23—C20	−7.0 (3)	C17—C22—C20—C21	3.7 (3)
V2—N3—C32—C31	46.0 (2)	C17—C18—C19—C21	3.4 (4)
V1—O2—C12—C9	−29.3 (3)	C24—C30—C29—C28	1.1 (3)
V1—O2—C12—C11	153.17 (16)	C24—C26—C27—C28	1.1 (4)
V1—N2—C14—C9	7.7 (3)	C13—N1—C15—C16	−137.4 (2)
V1—N2—C16—C15	22.8 (2)	C13—C4—C5—C6	175.5 (2)
V1—O1—C3—C4	10.4 (3)	C9—C12—C11—C10	1.9 (3)
V1—O1—C3—C2	−170.93 (17)	C9—C8—C7—C10	1.7 (4)
V1—N1—C15—C16	46.5 (2)	C7—C10—C11—C12	−0.5 (4)
V1—N1—C13—C4	−7.2 (3)	C7—C8—C9—C12	−0.2 (4)
O6—C30—C24—C25	−1.8 (3)	C7—C8—C9—C14	−176.6 (2)
O6—C30—C24—C26	−179.2 (2)	C20—C22—C17—C18	−3.0 (4)
O6—C30—C29—C28	−179.9 (2)	C20—C21—C19—C18	−2.6 (4)
O2—C12—C9—C14	−2.8 (3)	C2—C3—C4—C13	−172.5 (2)
O2—C12—C9—C8	−179.1 (2)	C2—C3—C4—C5	3.4 (3)
O2—C12—C11—C10	179.6 (2)	C2—C1—C6—C5	1.9 (4)
N4—C25—C24—C30	12.3 (4)	C23—N3—C32—C31	−137.4 (2)
N4—C25—C24—C26	−170.2 (2)	C23—C20—C21—C19	174.9 (2)
N4—C31—C32—N3	−42.8 (2)	C16—N2—C14—C9	−174.5 (2)
N2—C14—C9—C12	13.4 (3)	C5—C4—C13—N1	176.9 (2)
N2—C14—C9—C8	−170.3 (2)	C21—C20—C23—N3	176.9 (2)
O1—C3—C4—C13	6.2 (3)	C11—C12—C9—C14	174.7 (2)
O1—C3—C4—C5	−177.9 (2)	C11—C12—C9—C8	−1.6 (3)
O1—C3—C2—C1	177.6 (2)	C11—C10—C7—C8	−1.3 (4)
O4—C22—C17—C18	179.0 (2)	C27—C26—C24—C30	−0.8 (4)
O4—C22—C20—C23	5.9 (3)	C27—C26—C24—C25	−178.4 (2)
O4—C22—C20—C21	−178.4 (2)	C27—C28—C29—C30	−0.8 (4)
C14—N2—C16—C15	−155.1 (2)	C6—C1—C2—C3	1.1 (4)
C3—C4—C13—N1	−7.1 (4)	C29—C30—C24—C25	177.2 (2)
C3—C4—C5—C6	−0.6 (3)	C29—C30—C24—C26	−0.3 (3)
C25—N4—C31—C32	−155.7 (2)	C29—C28—C27—C26	−0.3 (4)
N1—C15—C16—N2	−43.4 (2)	C31—N4—C25—C24	−174.5 (2)
C4—C3—C2—C1	−3.7 (4)	C32—N3—C23—C20	177.0 (2)

### Hydrogen-bond geometry (Å, º)

**Table:**

*D*—H···*A*	*D*—H	H···*A*	*D*···*A*	*D*—H···*A*
C14—H14···O8^i^	0.93	2.48	3.368 (4)	159
C14—H14···O8*A*^i^	0.93	2.48	3.299 (7)	147
C25—H25···O7^ii^	0.93	2.51	3.391 (4)	158
C25—H25···O7*A*^ii^	0.93	2.66	3.457 (9)	144
C13—H13···O3^iii^	0.93	2.60	3.372 (3)	141
C23—H23···O5^iv^	0.93	2.59	3.364 (3)	141
O8—H8*A*···O2	0.82	2.12	2.926 (4)	167
O7—H7*A*···O6	0.82	2.18	2.940 (3)	153
O7—H7*A*···O4	0.82	2.64	3.246 (4)	132
O8*A*—H8*AA*···O1	0.82	2.18	2.984 (6)	166

Symmetry codes: (i) *x*, *y*+1, *z*; (ii) *x*, *y*−1, *z*; (iii) *x*−1/2, −*y*+5/2, *z*; (iv) *x*−1/2, −*y*+1/2, *z*.
