# Supplementary figures and images for: Crystal structure of {2,2′-[ethyl­enebis(nitrilo­methanylyl­idene)]diphenolato-κ4 O,N,N′,O′}oxidovanadium(IV) methanol monosolvate
Source: Acta Crystallogr Sect E Struct Rep Online. 2014 Oct 29;70(Pt 11):m380–1. doi: 10.1107/S1600536814023332 (PMC4257326; doi:10.1107/S1600536814023332)

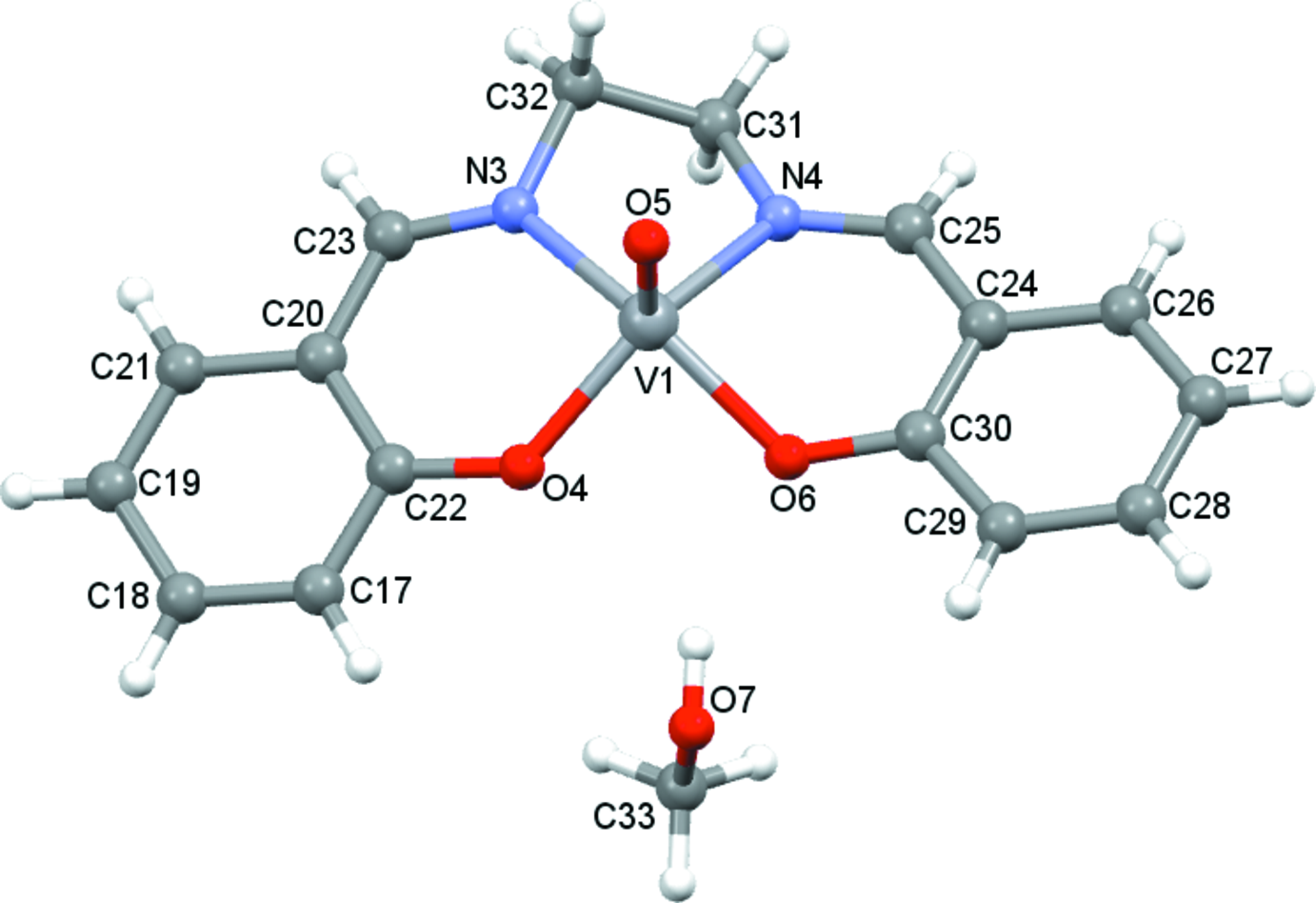

Supplement: Supplementary file 4 [file e-70-0m380-fig1.tif]
